# The Friendship Bench programme: a cluster randomised controlled trial of a brief psychological intervention for common mental disorders delivered by lay health workers in Zimbabwe

**DOI:** 10.1186/s13033-015-0013-y

**Published:** 2015-05-23

**Authors:** Dixon Chibanda, Tarryn Bowers, Ruth Verhey, Simbarashe Rusakaniko, Melanie Abas, Helen A Weiss, Ricardo Araya

**Affiliations:** Department of Community Medicine, Zimbabwe Aids Prevention Project, University of Zimbabwe, 92 Prince Edward Street, Harare, Zimbabwe; Institute of Psychiatry, Psychology and Neuroscience, King’s College London, London, UK; MRC Tropical Epidemiology Group, London School of Hygiene and Tropical Medicine, London, UK

**Keywords:** Randomised clinical trial, Depression, Common mental disorders, Task-shifting, Low-income country, Lay health workers

## Abstract

**Background:**

Common mental disorders (CMD) are a leading cause of disability globally. Emerging evidence indicates that in low and middle income countries the treatment gap for CMD can be addressed through the use of trained and supervised lay health workers (LHWs). Few clinical trials have evaluated the use of such task-shifting approaches in sub-Saharan Africa. In Zimbabwe, we have successfully piloted a task-shifting intervention delivered by LHWs. This protocol describes a cluster randomised controlled trial to assess the effectiveness of this intervention.

**Methods:**

Each of 24 randomly selected clinics from a pool of 42 in Harare will recruit 24 participants (N = 576). The clinics are randomised in a 1:1 ratio to receive either the intervention package [a problem solving therapy package delivered over a 4–6 week period by LHWs (N = 24) followed by a 6-week group support programme which focuses mainly on teaching a craft skill] or enhanced usual care, which includes usual care and psycho-education. Primary care attenders aged 18 years and above who score positive on a locally validated CMD screening questionnaire (Shona Symptom Questionnaire, SSQ-14) will be eligible for recruitment and asked for informed consent to participate in the trial. The primary measure is the SSQ score at 6 months.

**Conclusion:**

This effectiveness trial using LHWs to address the treatment gap for CMD will contribute to the body of knowledge on the feasibility and ability for scale-up of interventions for CMD.

**Trial registration:**

PACTR201410000876178.

## Background

Common mental disorders (CMD), which include depression, anxiety and somatoform disorders, are leading causes of disease burden globally [1]. In low and middle income countries (LMIC) CMD are poorly recognised and managed resulting in a large treatment gap [2]. Low cost packages of mental health care have been recommended for settings in sub-Saharan Africa, particularly stepped care approaches to managing depression [3]. This includes provision of simple psychological interventions, which can be delivered by non-specialists [4], and referral of those who do not recover. For this to be feasible for LMIC, task-sharing approaches need to be developed due to very low numbers of mental health professionals [5]. Currently psychological interventions for depression are virtually absent in government services in most low-income African countries [3, 6].

In recent years a renewed effort to address the treatment gap through task-shifting has shown promising results in a number of LMIC [7–9]. Task-shifting involves shifting basic responsibilities to non-specialist health professionals or paraprofessionals (nurses, social and community workers) in an attempt to reduce population burden and to release specialised health resources for more complex tasks [4, 10]. There is a growing body of evidence suggesting that non-health cadres, such as LHWs, can work effectively in addressing a wide range of public health issues including mental health [11].

In Chile, low-intensity low-cost treatments for depression delivered by LHWs have been successfully integrated into primary health care [7, 12]. These types of interventions include psycho-education, components of cognitive behavioural therapy (CBT), problem solving therapy (PST) and self-help approaches [8, 9, 11]. LHWs are available and affordable and, thus, appear to be one of the most appealing cadres to incorporate in the implementation of task-shifting interventions in LMIC. However, in accordance with the World Health Organisation (WHO) guidelines, it is imperative to ensure that they receive adequate training, supervision, support, career incentives and clear job descriptions to prevent overloading of these vital resources and undermining retention rates.

In Zimbabwe, where prevalence above 20% for CMD has been reported amongst adult primary care attendees [11, 13, 14], we recently piloted a task-shifting programme called The Friendship Bench and showed evidence of the feasibility and acceptability of using LHWs to deliver a psychological intervention for CMD [11]. The Friendship Bench programme consists of a cognitive behaviour therapy (CBT) based intervention that emphasises the use of problem solving therapy (PST) for the treatment of CMD. It is delivered by trained LHWs who are employed by the city health authorities in the city of Harare, Zimbabwe. The intervention consists of six sessions of 30–45 min of structured PST, delivered in a discrete area outside of the clinic building on a bench (The Friendship Bench). The PST components consist of problem listing and identification, problem exploration, developing an action plan, implementation, and follow up. We have found preliminary evidence of a clinically meaningful improvement in CMD using this locally adapted PST approach. In a cohort (n = 320) of patients recruited at primary health care facilities the mean score of a locally validated screening tool for CMD, the Shona Symptom Questionnaire (SSQ-14) [15] fell from 11.3 (SD 1.4) before treatment to 6.5 (SD 2.4) after 3–6 sessions on the Friendship Bench [11]. However, despite these promising results, there have been no effectiveness trials of the intervention conducted to date.

This protocol describes the design of a cluster randomised controlled trial of the Friendship Bench across 24 clinics in the city of Harare’s Health Department. The study population will include all adults visiting the local clinics for HIV and non-HIV related health problems who are able to give written consent and meet study inclusion criteria.

The aim of this trial is to compare the Friendship bench intervention with an enhanced usual care component. We hypothesize that the Friendship Bench intervention will be superior to the enhanced usual care for CMD in the study population in decreasing depression severity and prevalence 6 months after treatment.

## Methods/design

Preliminary work leading to the current protocol consisted of a systematic review of psychological interventions for CMD in LMIC [16]. Further work included the identification, translation, back-translation and validation of study tools; working in partnership with the Harare City Health department to establish terms of engagement, to conduct a descriptive evaluation of all their clinics, and to administer an assessment of all 300 LHWs employed by this department through qualitative interviews with the aim of determining core competencies of this cadre. Included in the preliminary work was the development of a technological platform using cloud computing to support the LHWs through task-shifting, as well as for the collection and storage of data. Prior to this, five focus group discussions and six in-depth individual interviews were conducted with the LHWs, and six in-depth interviews were carried out with clients in order to gain their perspectives of having delivered or received the intervention. Findings from this qualitative work informed the adaptation of the intervention for the trial [17].

The intervention will be delivered by trained LHWs, supervised and supported by their existing immediate supervisors, the District Health Promotion Officers (DHPOs), who will be contacted in the event that a client presents with a red flag, such as very high scores on the assessment tool (the SSQ-14 [15]) or suicidality. The DHPOs in return will get support from the clinical psychologists and psychiatrists. All DHPOs have university education at masters level in either social sciences, public health or health promotion. The DHPOs will receive a 2 months training on CMD, use of screening tools, and will participate in the pilot trial and training of their LHWs.

### Design

The study is a cluster randomised controlled effectiveness trial with a 6-month follow-up. It will be conducted in 24 of the busiest primary care clinics, selected from the 42 clinics across Harare, Zimbabwe. All 24 clinics were eligible for selection based on a formal randomisation process attended by all City Health clinic senior staff members. Prior to randomisation, the 24 clinics were grouped into 5 strata based on HIV status, density of housing, clinic size and gender distribution of clinic users. This stratification resulted in 112,000 possible allocations for allocating clinics between the two arms in a 1:1 ratio within stratum, and of these, 3,268 allocations satisfied further criteria to ensure balance on HIV prevalence, clinic size, staff size and gender. An official public randomisation exercise facilitated by the Director of Health Services was carried out by city health staff who were not involved in the study before commencement of the trial to select one allocation. The entire study team was blinded to this exercise.

### Setting

The study is being conducted in Harare, Zimbabwe. As of 2014 there were 42 primary health care clinics in operation around Harare with each one catering for 20,000–80,000 people, from the most socio-economically disadvantaged sectors of the population.

### Recruitment procedure

All adult persons attending primary health care facilities will be sensitised verbally by a study team member who will initially inform all those waiting to be seen on the day about the study by explaining what common mental disorders are and how they can affect existing medical conditions such as hypertension, HIV, TB, diabetes. After the verbal sensitisation procedure patients will be allocated a computer generated random number corresponding to their position in the waiting list for the day. All those allocated the random number will be invited for a more detailed talk about the study before being invited to participate in the next stage of the recruitment. Those showing interest will immediately be screened and assessed for study eligibility as described below.

### Screening and assessment instruments

1. Common mental disorders (CMD) will be measured by the SSQ-14 which has been used extensively in Zimbabwe as a screening tool with reliable sensitivity and specificity [15]. The SSQ-14 is a dichotomous 14-item questionnaire which will be administered by a research assistant who is blinded. The SSQ-14 is a tool for detecting CMD used largely at primary health care settings in Zimbabwe. Until recently the cut-off score for this tool was 8/14, however, following the re-validation of this tool in an HIV setting the cut-off score has been raised to 9/14. The SSQ-14 will be the primary outcome measure for CMD at 6 months after recruitment.
2. Clinical diagnosis of depression will be measured by the Patient Health Questionnaire (PHQ-9).

The PHQ-9 is a 9-item Likert scale which is used to make a diagnosis of depression [18]. This tool has recently been validated in Zimbabwe and the cut-off score for severe depression has been set at 20/27 following the piloting of the tool amongst patients utilising primary health care facilities. The PHQ-9 will be used to screen for depression and establish severity of symptoms in all participants who score above 11 on the SSQ-14 as a way of confirming red flags (suicidal cases) and the need to refer to the next level of care. Furthermore, it will be used as a secondary outcome measure for depression at 6 months because the SSQ-14 is not a specific measure for depression. All participants in the intervention arm scoring ≥20 on the PHQ-9 will be immediately referred to a clinical psychologist for further assessment. Participants referred for further care will not be excluded from the study but will receive the extra input from either a psychiatrist or clinical psychologist in addition to the PST, while in the control arm individuals will be referred to a tertiary facility offering psychiatric services as part of standard care in the event that the clinic staff are unable to manage the cases. Depression as defined by the PHQ-9 using a cut point of 11 and above for caseness [18] at 6 months after entering the trial will be our secondary outcome measure.

### Inclusion criteria

All persons residing in the area in possession of a valid national identification card attending the local clinics who are aged 18 and above and are able to give written informed consent will be eligible for enrolment if they score above the pre-determined cut-off point (≥9) on the SSQ-14 [15].

### Exclusion criteria

All persons who are unable to comprehend the nature of the study in either English or *Shona* (local language), have suicidal intent, end stage AIDS, are currently in psychiatric care, present with current psychosis, intoxication, and/or dementia will be excluded. All those excluded for medical reasons will be referred for appropriate care to one of two tertiary facilities in Harare. Those reported to be physically unwell by the clinic Nurse-in-Charge will be excluded. Pregnant women in the third trimester and women within the 3-months post delivery period will be excluded. All those not residing in the geographical locality or whose address can not be verified through the clinics’ registry will also be excluded.

To ensure that all clinic attendees have an equal chance of being selected, three participants will be recruited per weekday in those clinics that have fixed days allocated for specific medical conditions such as HIV/AIDS, diabetes, hypertension. In clinics with no such medical condition-based services we will recruit participants until our total of 24 per site is reached, ensuring that the corresponding strata in the control sites receive the same approach.

### Sample size

The sample size of 24 clusters, each with 24 participants enrolled provides 80% power to detect an effect size of 0.75, assuming a coefficient of variation (k) = 0.2 and 20% loss to follow-up. We would have 90% power to detect this effect size if the coefficient of variation is smaller (k = 0.16), and 90% power to detect a larger effect size of 0.85 if k = 0.19. The coefficient of variation is defined as the between-cluster variation divided by the mean value, for the outcome of interest (mean SSQ score). In the absence of this measure for each clinic, we based this on the value of k for HIV prevalence. The effect size is based on a recent systematic review, which identified six intervention trials of lay-health worker (LHW) led interventions with severity of CMD as an outcome [4]. The pooled effect size for the LHW intervention vs. control at 6 months was 0.75 (95% CI 0.21–1.29) [4].

### Intervention

The intervention consists of six sessions of a PST package which is delivered on a bench in a discrete area outside of the local clinic [11]. Each session will last approximately 30–45 min with the first session lasting up to an hour (Table 1). The 6 sessions will be completed within a 4–6 week period. Supervisors will not be present during the actual sessions but they will have access to the audio-recording after each session. All sessions will be audio recorded for fidelity, with fidelity being assessed using a checklist to ensure that the LHW has covered all the critical PST components. The fidelity assessment will be carried out by the study clinicians, which include psychiatrists and clinical psychologists. LHWs will have access to immediate support through either their immediate supervisor who will be available at clinic level should the LHW require assistance during a session, a study clinical psychologist, the study coordinator or through the use of a mobile phone device to enable instant communication with team members when nobody is available at clinic level. The psychological approach is based on providing psycho-education (information, advice and support) together with a problem-solving module that includes a component of positive activity scheduling (behavioural activation). In addition, patients will receive up to 6 brief text messages and/or calls reinforcing the PST approach and encouraging adherence to treatment as our survey of patients utilizing primary health facilities indicates that over 90% possess a mobile phone. The small proportion of participants who are recruited for the study but have no mobile phone will be asked to provide a mobile phone of an alternative relative or friend. These text messages will be sent to participants who attend less than 3 sessions or those who the LHW and supervisor feel need extra support. Text messages will be sent out once a week. Whenever necessary LHWs will be encouraged to act as facilitators to help patients to get access to other sources of support using their mobile devices. The income generating component will be open to all persons assigned to the intervention arm if they chose to after receiving a minimum of four sessions of the PST. This income generating component will focus on group peer support and sharing while actively crocheting a shopping bag from recycled plastic materials, the latter being a skill that can generate income for the participants. This forum will give participants an opportunity to learn through behaviour activation.

**Table 1:** Description of the problem solving therapy intervention

Theoretical basis	Cognitive behavioural therapy
Delivering agent	Lay health workers (Health promoters). Mean age 58, all female, mean years of education 8, previous training in home based care for people living with HIV & AIDS, in community follow-up of persons on TB treatment and in delivering community health education and promotion e.g., through encouraging immunisation and methods to control disease outbreaks
Structure of intervention	Six weekly sessions of 30–45 min delivered through the Friendship Bench over 6 weeks, including home visits where deemed necessary. The first session lasting between 45 and 60 min
Structure of sessions and areas covered	*Part 1*. **Problem identification (Kuvhura pfungwa)**: (A) Share Shona Symptom Questionnaire (SSQ) information with client, explain symptoms in relation to *kufungisisa*, (B) Actively listen to clients story, identify and list problems raised, clearly define problem/s. **Problem exploration (kusimudzira)**: (C) understand the story, help client prioritise problems by summarizing and asking if you have missed anything, (D) brainstorm practical/feasible solutions, outline the options available (*these have to come from client*), encourage client to think over solutions of each problem before having the client decide which one to focus on. Help client to come up with a specific, measurable, achievable and realistic solution (*don’t tell client what to do*). Agree what the client will do before you next meet and set appointment date *Part 2*. **Reassure** **(Kusimbisa)** (E) Home visit if needed before second meeting, otherwise see again on the bench, how did it go? Went well, then reassure praise encourage. If no progress or new obstacles present then go back to Part 1 contents, redefine problem and goals, what were the obstacles? Problem solve around obstacles and give homework again and reassure, you can phone or send SMS to reassure client *(up to 6 per client).* *Part 3* **(kusimbisisa)**. (F) Summarise session 1, how did it go? Going well then reassure and ecourage. Still having problems with agreed plan? Go back to Part 1 again or if you feel frustrated go to supervisor
Remember	**Action plan: (**G) Zero in on a specific solution, focus on what client wants to do and *not what you think should be done*, (H) How, when, what assistance is needed? Referral if necessary (I). Identify activities the person used to find rewarding and which matter to them and encourage these (J), **Implementation:** (K) How will it be done? Motivate; homework, Refer after 4th session to support group. **Follow up: (**L) What has been achieved? What were/are the obstacles if any? Go back to Part 1 as often as needed during the 6 sessions (M) Reinforce. What has been achieved, repeat SSQ score. (N) No improvement refer to supervisor. nurse counsellor
Tools	SSQ-14, Friendship bench manual, referral protocol
Supervision	Weekly group supervision by a clinician (Psychologist) or senior study team member trained in PST. Access to direct mobile voice call to support team

LHWs will receive supervision and support from the clinical team at site level or through mobile phones using voice calls, and where necessary SMS messaging. The supervision will be for critical cases such as those with suicidal ideation who may need the input of a psychiatrist/psychologist and or medication. The SMS messaging/voice call will also be sent by the LHW to the participant particularly in cases where a participant has not turned up for a session on the bench and where challenges such as being unable to contact participant are encountered the project coordinators with the LHW will follow up with a voice call and if this yields no results a physical home visit will be carried out by the two. The support structure is based on a pre-determined algorithm developed during the formative research. This consists of study screen tool cut-off scores, criteria for referral including assessment for “red flags” clients who are suicidal and procedure immediate referral to a tertiary facility.

The control group will receive enhanced usual care (EUC) care through the clinic which will include psychoeducation on CMD, medication if indicated and/or referral to a psychiatric facility as part of usual standard procedure when clinic staff are unable to manage serious CMD cases. Participants will also receive between 2 and 3 supportive SMS messages or voice calls with the last SMS message or voice call being a reminder to attend the 6-months assessment.

### Data management

Data will be collected using tablet computers, all data is consecutively uploaded to a cloud enabling easy data handling for statistic purposes. The computing system is password protected, encrypted and only accessible to authorised study team members, any access to the system is automatically recorded. Data will be managed according to International Conference on Harmonisation guidelines for Good Clinical Practices. Participants will receive a unique study identification number recorded on all forms. Numbers will be maintained by the Project Director on a password protected, secure computer. Data will be entered into a password-protected database with independent double-data entry and a third person to resolve discrepancies. To minimize errors, range checks and skip patterns within data entry screens will be used. Database files will be exported in SAS format for bi-weekly batch error checking, monthly reporting and analysis. Data will be backed-up on an external hard-drive weekly. Recorded tapes will be transcribed into electronic records which will be backed-up on an external hard-drive after each session. All data will be kept confidential, under lock-and-key, accessible only to trained study staff. Participants’ data will be identified by an ID number only, and a link between names and ID numbers will be kept separately under lock-and-key.

### Data analysis

Data will be exported to Stata 13.1 for analysis. Baseline comparability will be assessed for individuals who did not consent to be part of the trial, and of participants who did not complete outcome assessments. Comparability of participants in the two arms will be assessed for potential confounding factors, notably: age, sex, HIV status and SSQ score. Due to the relatively small number of clusters, analyses will be based on cluster-level summary measures, as individual-level regression methods do not perform robustly when there are relatively few clusters per arm, especially for stratified cluster randomized trials [19]. For the primary outcome (SSQ score), the mean SSQ score for each cluster will be calculated and shown by strata and arm. The arithmetic mean and SD of these mean scores and associated 95% CI will be estimated by arm. Linear regression of the mean score on strata and arm (2-way analysis of variance (ANOVA) on arm and strata) will be used to estimate the difference in SSQ score and 95% CI associated with the intervention. For the binary outcome (proportion with depression), the risk for each cluster will be calculated, and shown by strata and arm. The mean and SD of the log risk will be used to estimate the geometric mean and associated 95% CI for each arm of the study. Linear regression of the log mean risk on strata and arm will be used to estimate the risk ratio and 95% CI. Linear regression of the mean risk on strata and arm will be used to estimate the risk difference and 95% CI. The approximate variance for the mean risks will be obtained based on the residual mean square from a 2-way ANOVA on arm and strata. A 95% CI for this will be calculated from the variance using a t-statistic with 18 degrees of freedom. Pre-defined sensitivity analyses includes adjustment for baseline SSQ score and other factors with imbalance (e.g. age, sex, HIV prevalence). We will also include tests for effect-modification by gender and other key factors.

### Ethical issues

The study will be conducted according to regulations, guidelines and principles that have been endorsed at local and international levels. The protocol was approved by the Medical Research Council of Zimbabwe (MRCZ), Kings College London (KCL), and London School of Hygiene and Tropical Medicine (LSHTM).

## Discussion

The funding provided through Grand Challenges Canada has provided a unique opportunity to carry out a cluster randomized controlled trial of this promising intervention which has been running since 2006. The use of LHWs to address the treatment gap for mental, neurological and substance use disorders (MNS) particularly in LMIC has been recommended by the WHO [20]. Evidence from several LMIC indicates that this is feasible and cost-effective [7–9]. There is, however, a dearth of data on controlled trials in sub-Saharan Africa [21, 22]. This study will contribute to the much needed body of knowledge on the efficacy of psychological interventions delivered by LHWs in LMIC. Furthermore, the inclusion of a technological platform for both data collection and storage, as well as for support of the LHWs delivering the intervention, will give an opportunity to objectively assess the feasibility of supporting community health workers via e-health packages. With HIV now considered a chronic disease this trial will also look at the feasibility of using LHWs to address the issue of co-morbidity between HIV and MNS an approach that has been widely encouraged recently [23]. We hope to gain insight on this during analysis of our data as we expect to have a large population of PLWH in the study. Our study will be of interest to researchers involved in the development of psychological interventions for CMD in similar LMIC settings. It can also provide information on the feasibility of utilising the task-shifting approach promoted by the WHO.

Strengths of the study include the integration of three components (health, technological and income-generation) and the use of LHWs who are already integrated in a primary health care system that focuses on both communicable and non-communicable diseases. Moreover, the study will be innovatively addressing some of the most challenging barriers to mental health services, such as human resources, quality services, access to care facilities and medication as well as poverty. If successful, the results can be potentially scaled up to make a major contribution to improving access to treatment for CMD in Zimbabwe and secondly, it could possibly be adapted and applied across countries in the region. Weaknesses of the study include lack of blinding of some of the study team members, as is usual in a cluster randomised trial, particularly for the study coordinators who will actively be involved in the recruitment process although they will not play a role in the collection and assessment of outcome measures. Furthermore, the exclusion of people not residing in the geographical area of the clinic and those without valid national identification cards may exclude potential participants, however, this approach will minimise contamination and loss to follow up.

This trial offers an opportunity to show the effectiveness of a lay health worker driven intervention to address the treatment gap for CMD in LMIC. With screening and treatment of CMD at primary care level becoming routine, recognition of these problems will increase and effective help will potentially be made accessible, thereby reducing suffering and decreasing referral to tertiary level.

## Abbreviations

CBT: cognitive behavioural therapy
CMD: common mental disorders
EUC: enhanced usual care
HIV/AIDS: human immunodeficiency virus and acquired immune deficiency syndrome
LHW: lay health worker
LMIC: low- and middle-income countries
MNS: mental neurological and substance use disorders
PHQ: Patient Health Questionnaire
PST: problem-solving therapy
SSQ-14: Shona Symptoms Questionnaire 14
